# Network science characteristics of brain-derived neuronal cultures deciphered from quantitative phase imaging data

**DOI:** 10.1038/s41598-020-72013-7

**Published:** 2020-09-15

**Authors:** Chenzhong Yin, Xiongye Xiao, Valeriu Balaban, Mikhail E. Kandel, Young Jae Lee, Gabriel Popescu, Paul Bogdan

**Affiliations:** 1 Ming Hsieh Department of Electrical and Computer Engineering, University of Southern California, Los Angeles, CA 90007 USA; 2 Department of Electrical and Computer Engineering, Beckman Institute for Advanced Science and Technology, University of Illinois at Urbana-Champaign, Champaign, IL 61801 USA; 3 Neuroscience Program, University of Illinois at Urbana Champaign, 208 N Wright St., Urbana, IL 61801 USA

**Keywords:** Neuroscience, Mathematics and computing

## Abstract

Understanding the mechanisms by which neurons create or suppress connections to enable communication in brain-derived neuronal cultures can inform how learning, cognition and creative behavior emerge. While prior studies have shown that neuronal cultures possess self-organizing criticality properties, we further demonstrate that in vitro brain-derived neuronal cultures exhibit a self-optimization phenomenon. More precisely, we analyze the multiscale neural growth data obtained from label-free quantitative microscopic imaging experiments and reconstruct the in vitro neuronal culture networks (microscale) and neuronal culture cluster networks (mesoscale). We investigate the structure and evolution of neuronal culture networks and neuronal culture cluster networks by estimating the importance of each network node and their information flow. By analyzing the degree-, closeness-, and betweenness-centrality, the node-to-node degree distribution (informing on neuronal interconnection phenomena), the clustering coefficient/transitivity (assessing the “small-world” properties), and the multifractal spectrum, we demonstrate that murine neurons exhibit self-optimizing behavior over time with topological characteristics distinct from existing complex network models. The time-evolving interconnection among murine neurons optimizes the network information flow, network robustness, and self-organization degree. These findings have complex implications for modeling neuronal cultures and potentially on how to design biological inspired artificial intelligence.

## Introduction

Current research in neuroscience models the brain as a dynamic complex network whose connections change continuously as we advance through life^1,2^. Consequently, there is significant motivation for understanding the mechanisms by which neurons create or suppress connections to enable hierarchical parallel processing in the brain and explaining how learning, cognition and creative behavior emerge^3–8^. Moreover, the brain connections are thought to obey a constrained optimization, such as maximization of information processing capacity (efficiency) while minimizing the energy expenditure^9^.

Motivated by these challenges, there is a growing effort on analyzing the evolution and emergence of connectivity and its implications for information processing in both in vitro neural cultures and live brain sensing. For instance, prior efforts on investigating the morphological evolution of assemblies of living neurons showed that cultured neurons self-organize and form complex neural networks that exhibit a small-world structure (a network with many highly interconnected clusters with few long-range connections among clusters)^10^. Moreover, Okujeni et al.^11^ investigated the impact of neuron clustering (by modulating the protein kinase C) on the spontaneous activity in neuronal culture networks and showed that higher clustering contributed to synchronous bursting in some parts of the neuronal culture networks. Besides analyzing neuronal cultures at the macroscale and mesoscale, pioneering efforts that combined functional magnetic resonance imaging (fMRI) based on blood oxygen level–dependent (BOLD) contrast with bulk calcium indicator signal measurement enabled to investigate in vivo the neuronal and glial activity coupling in rat somatosensory cortex^12^.

In this study, relying on label-free quantitative microscopic imaging of neurons (with a resolution of 10000 times higher than MRI technology), we reconstruct the neuronal culture networks (constructed from rat neurons) and neuronal culture cluster networks (constructed from mice neurons) and analyze their topological properties in order to elucidate how neurons generate new connections and connect with each other over time. In the brain-derived neuronal culture network, the somas and the neurites represent the nodes and the edges, respectively. In the brain-derived neuronal culture cluster network, the neuronal clusters and the cluster neurites (between two different clusters) represent the vertices and their corresponding connections. By analyzing the structure and evolution of neuronal culture network and neuronal culture cluster networks, we demonstrate that these neuronal culture networks exhibit a unique self-optimization and assortative connectivity behavior, as well as a peculiar multifractal structure that cannot be captured by existing complex network models^13–16^. These findings suggest that a new class of mathematical models and algorithmic tools need to be developed for describing the interwoven time-varying nature of the neuronal culture’s information processing as well as for understanding how these dynamic networks are controlled, or explaining the mechanisms of spontaneous activities in neuronal culture networks evolution.

**Figure 1:**
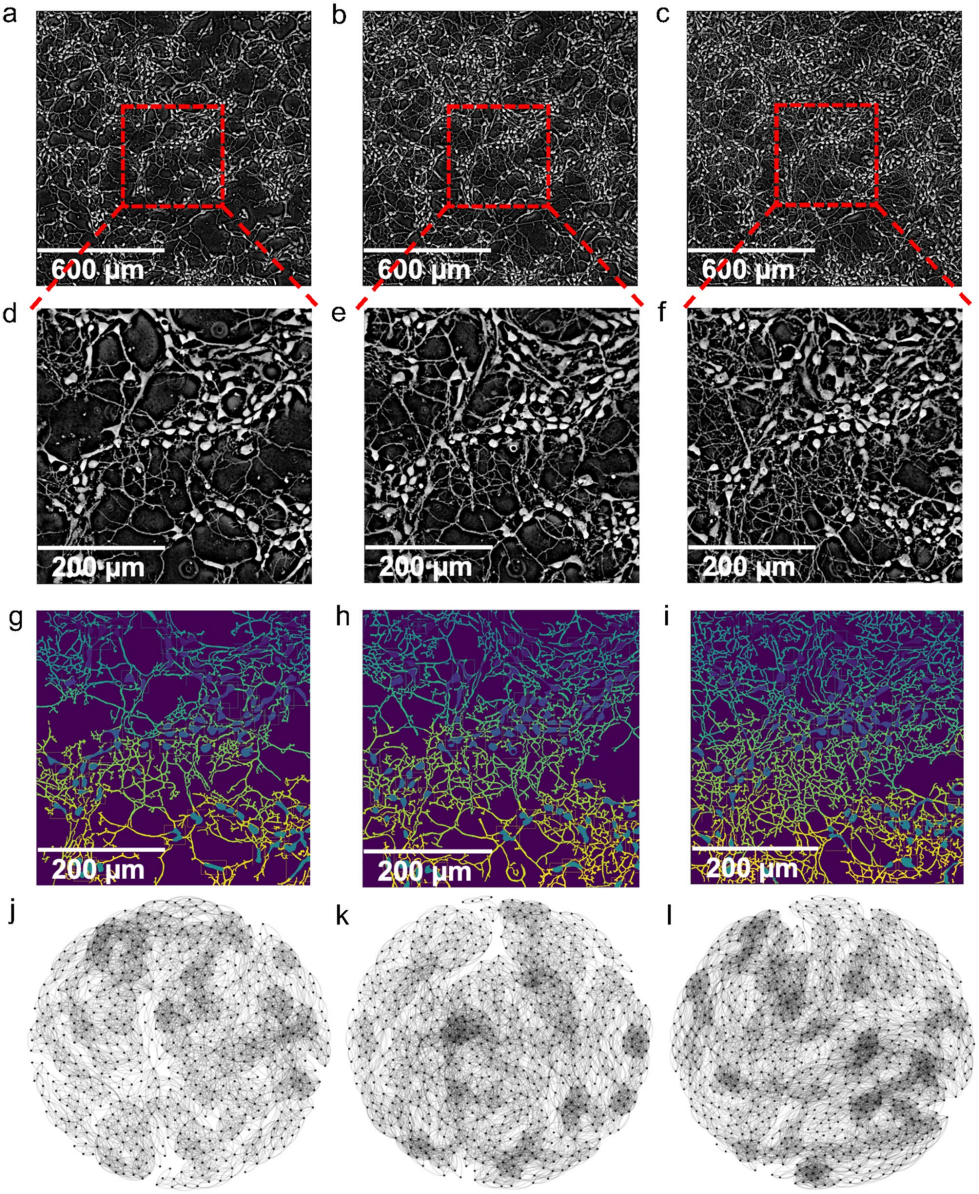
Layouts for neuronal culture networks at three representative time points. Neurons at the start of the experiment at time t = 0 h (**a**), t = 7 h (**b**) and the end of the experiment t = 14 h (**c**). The magnification zoom of the neurons at t = 0 h (**d**), t = 7 h (**e**) and t = 14 h (**f**). (**g**–**i**) show the identified neurons and their connections obtained with our algorithm (see “Methods” section on “Cell segmentation and neural tracing”) for the three corresponding time points (each neuron and neurite is identified by a unique color). After constructing the adjacency matrices from the tracing and segmentation algorithm, the visualization of the network layouts at t = 0 h (**j**), 7 h (**k**) and 14 h (**l**) are presented.

## Results

### Neuronal interconnection dynamics displays assortative behavior

**Figure 2:**
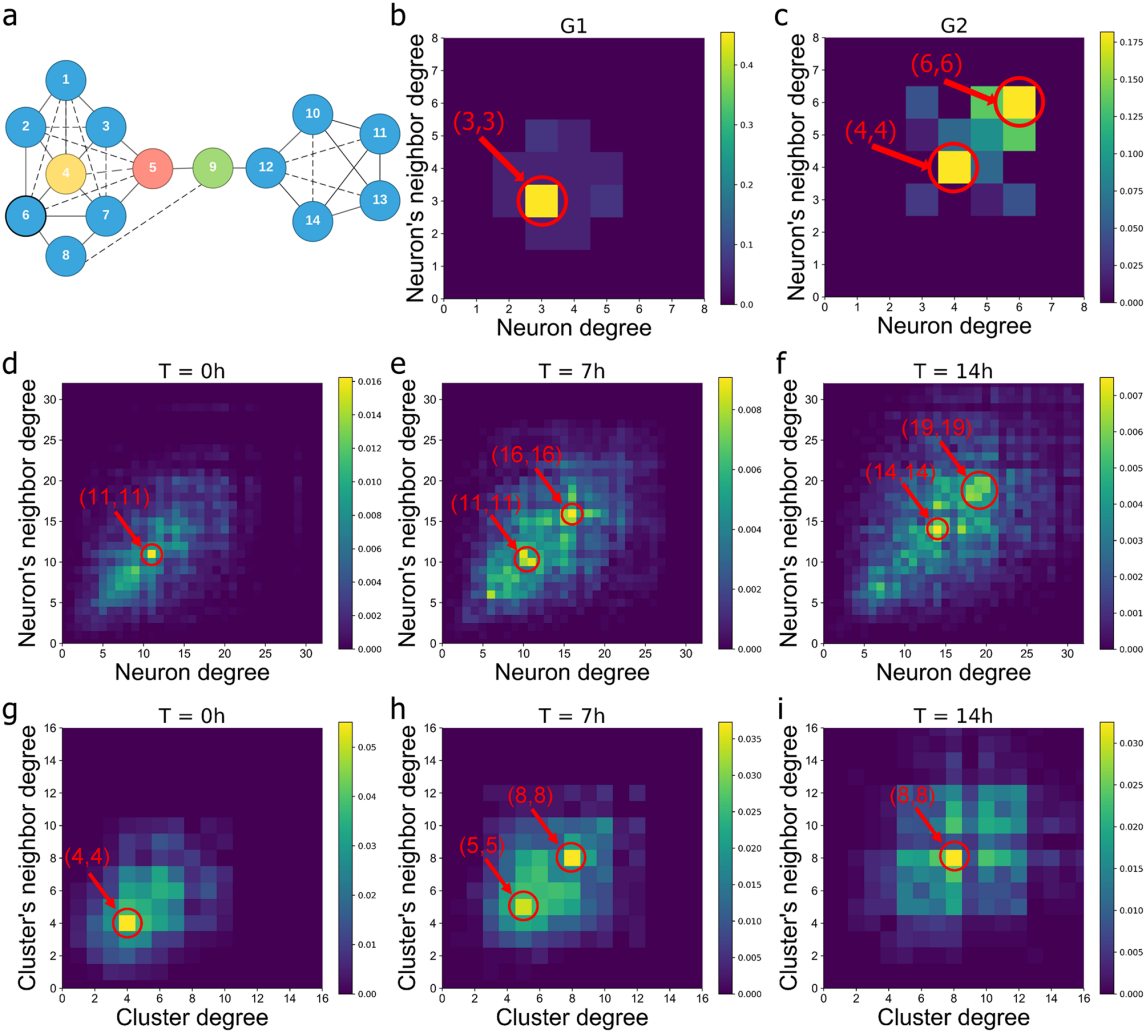
Degree distribution for neurons and neural clusters. An artificial network example (**a**), where the yellow node has the highest degree centrality, the red node has the highest closeness centrality and the green node has the highest betweenness centrality (with solid lines and dotted lines). The node-to-node degree distribution for the network example (**a**) without (**b**) and with (**c**) additional dotted lines in order to mimic the connectivity phenomena observed in neural cluster networks, where the color bars present the occurrence frequency of the node-to-node matrices and the red cycles with connection pairs represent the coordinate of the peak values in the matrix. The node-to-node degree distribution for the neuronal culture networks at the start of the experiment t = 0 h (**d**), after 7 h (**e**), and the end of the experiment after 14 h (**f**). The node-to-node degree distribution for the neuronal culture cluster networks at the start of the experiment t = 0 h (**g**), after 7 h (**h**), and at the end of the experiment after 14 h (**i**).

In this paper, we investigate neuronal culture networks (constructed from rat neurons) and neuronal culture cluster networks (constructed from mice neurons). We firstly present the generated neuronal culture networks’ layouts in Fig. 1 (Supplementary Fig. S1). Figure 1a–c present the neurons images from the SLIM imaging experiments (for details on SLIM imaging experiments see “Methods” section “Sample preparation” and “Microscopy”), Fig.1d–f show the zoomed portion of the middle region of the neuron images, Fig. 1g–i present the neuronal culture networks’ layouts after executing our tracing algorithm (“Methods” section “Cell segmentation and neural tracing” provide detailed information). Different colors represent the different identifications for each neuron and neurite. Figure 1j–l consists of a network representation of the neurons with their spatial position of neurons altered. In addition, we also analyze neuronal clusters obtained through a similar procedure as in Teller et al.^17^ while using a higher resolution provided by SLIM imaging which decouples amplitude artifacts from highly detailed cellular information. The neural computation emerges from the complex dynamic interconnection patterns and signaling among neurons. Consequently, to decode the complexity of the dynamic neuronal interconnections, we investigate first the node-to-node degree distribution. While the degree distribution^18^ captures the first-order statistic of a complex network, the node-to-node degree distribution offers second-order statistical information and explains how a node of a specific degree connects to lower or higher degree nodes. To study the second-order statistics of the networks of neurons and neuronal clusters, we consider three consecutive snapshots (i.e., after 0, 7, and 14 h) and estimate for each target node the degree distribution of its neighbors. For example, if a neuron with degree 5 connects with another one with degree 4, we add 1 on the coordinate (5,4) in the 2D node-to-node degree distribution plot. Figure 2a illustrates an artificially generated network example. The dotted lines represent new connections to this artificial network after a period of time. Figure 2b,c illustrate the node-to-node degree distribution for the artificial network example without and with the dotted links, respectively. In Fig. 2d–i, the x-axis represents the neuron degree and the y-axis represents the degree of its neighbor. The rationale for constructing the node-to-node degree distribution plot is twofold: (i) In each separate graph, we can find the tendency of a neuron with a certain degree to connect to neurons with lower, the same or higher degree; (ii) In the neuronal culture networks, the degree varies due to informational exchanges over the new neural connections. The length of the neurites grows over time. However, a neurite cannot be recorded as an edge in our network before its axon terminal connects with another neuron. Investigating the degree distribution at different time points can help us learn how the neurites grow and how neuronal culture networks construct new connections.

By analyzing the node-to-node degree distribution for the network of neurons (i.e., Fig. 2d–f) and the network of neuronal clusters (i.e., Fig. 2g–i) across time, we observe: (i) In the beginning (t = 0 hours), based on Fig. 2d,g, the network of neurons and the network of neuronal clusters display a preferential attachment (PA)^19^ phenomenon in the sense that neurons or clusters tend to connect to nodes of the same degree. In the network of neurons, the most frequent connection pattern corresponds to neurons of degree 11 that connect also to neurons with 11 connections. In the network of neuronal clusters, the most frequent connection pattern corresponds to nodes with 4 links that also connect to nodes of the same degree. (ii) After 7 h, from Fig. 2e,h, we observe a discrepancy between the neuronal culture network and the neuronal culture cluster network. The network of neurons displays three peaks corresponding to the following cases: neurons of degree 16 tend to connect to other neurons of degree 16, neurons of degree 10 connect to other neurons with degree 11, and neurons of degree 11 connect to other neurons of degree 10. Also, the network is evolving and displays an increasing connectivity. In contrast, the neuronal culture cluster network exhibits only two peaks: the neuronal clusters (nodes) of degree 5 connect to other nodes of degree 5 and nodes of degree 8 connect to other clusters of degree 8. (iii) After 14 h, from Fig. 2f,i, the network of neurons displays a two peak pattern (i.e., nodes of degree 14 are more likely to connect to nodes with degree 14 and nodes of degree 19 also prefer to connect to other nodes with degree 19) and the network of neuronal clusters shows a single peak (i.e., the nodes of degree 8 are more likely to connect with other nodes with degree 8). We also observe from all these plots that the distribution of neurons and their neighbors’ degree has a higher density along the diagonal. One can explain this phenomenon by the existence of multiple communities of neurons within which each neuron is likely to be fully connected with its community members (Fig. 1j–l); consequently, most of the neurons within the same community can have a similar degree. Furthermore, we can observe from all plots in Fig. 2 that the regions with a higher occurrence frequency of node-to-node distribution move and spread along the diagonal as time goes on. This implies that over time more edges are generated and nodes gain in degree. We conclude that a node has a high probability of connecting to other neurons that have the same degree. The assortativity coefficient represents the tendency for nodes to connect to other nodes with similar properties within a network^17^. Thus, we calculate the assortativity coefficient of a single neuronal culture network and a single neuronal culture cluster network for three consecutive snapshots in Table 1. Based on Table 1, all the assortativity coefficients are positive values and both neuronal and neuronal culture cluster networks have a decreasing tendency of assortativity coefficient. Based on Fig. 2 and Table 1 results, we conclude that (i) a neuron has a high probability of connecting to other neurons that have the same degree and (ii) neurons will set up new connections with neighboring neurons over time which have proximate degrees.

**Table 1:** The assortativity coefficient for neuronal culture networks and neuronal culture cluster networks in consecutive snapshots.

Models	Assortativity		
	Start (t = 0 h)	Middle (t = 7 h)	End (t = 14 h)
Neuronal culture network	0.5118	0.4400	0.4258
Neuronal culture cluster network	0.3153	0.2663	0.2109

### Microscale neuronal culture networks and mesoscale neuronal culture cluster networks optimize the network information transfer (flow) and robustness

Neural computations governing the sensorial processing, perception, and decision-making emerge from the information transfer across interwoven time-varying complex neuronal culture networks^20^. To investigate the performance of information transfer from biological data consisting of only snapshots of microscale neuronal culture networks and mesoscale neuronal culture cluster networks, we quantify their degree centrality, the closeness centrality and betweenness centrality^21^. Generally speaking, the centrality measures the importance of a node across a heterogeneous complex network. For instance, in a social network, the influencer nodes (e.g., politicians, TV stars) have a large number of followers and hence are capable of propagating specific messages faster than other network nodes. The degree centrality measures the number of links incident upon a node and can be related to the localized network transport or throughput capacity. The closeness centrality of a node quantifies the average length of the shortest path between the target node and all other nodes in the graph and encodes information about the information transmission latency across a specific network topology. The betweenness centrality measures the number of times a node appears along the shortest path between all pairs of two nodes. The higher the betweenness centrality of a node, the more information paths pass through it and the less robust the network is to targeted attacks on this node (for details on the degree-, closeness-, and betweenness-centrality, see “Methods” section “Networks centrality”). Fig. 2a shows an artificial network example (with additional connections) where the red node has the highest closeness centrality, the yellow node has the highest degree centrality, and the green node has the highest betweenness centrality (Supplementary Tables S1, S2 exhibit the degree-, closeness-, and betweenness centrality for each node in Fig. 2a).

**Figure 3:**
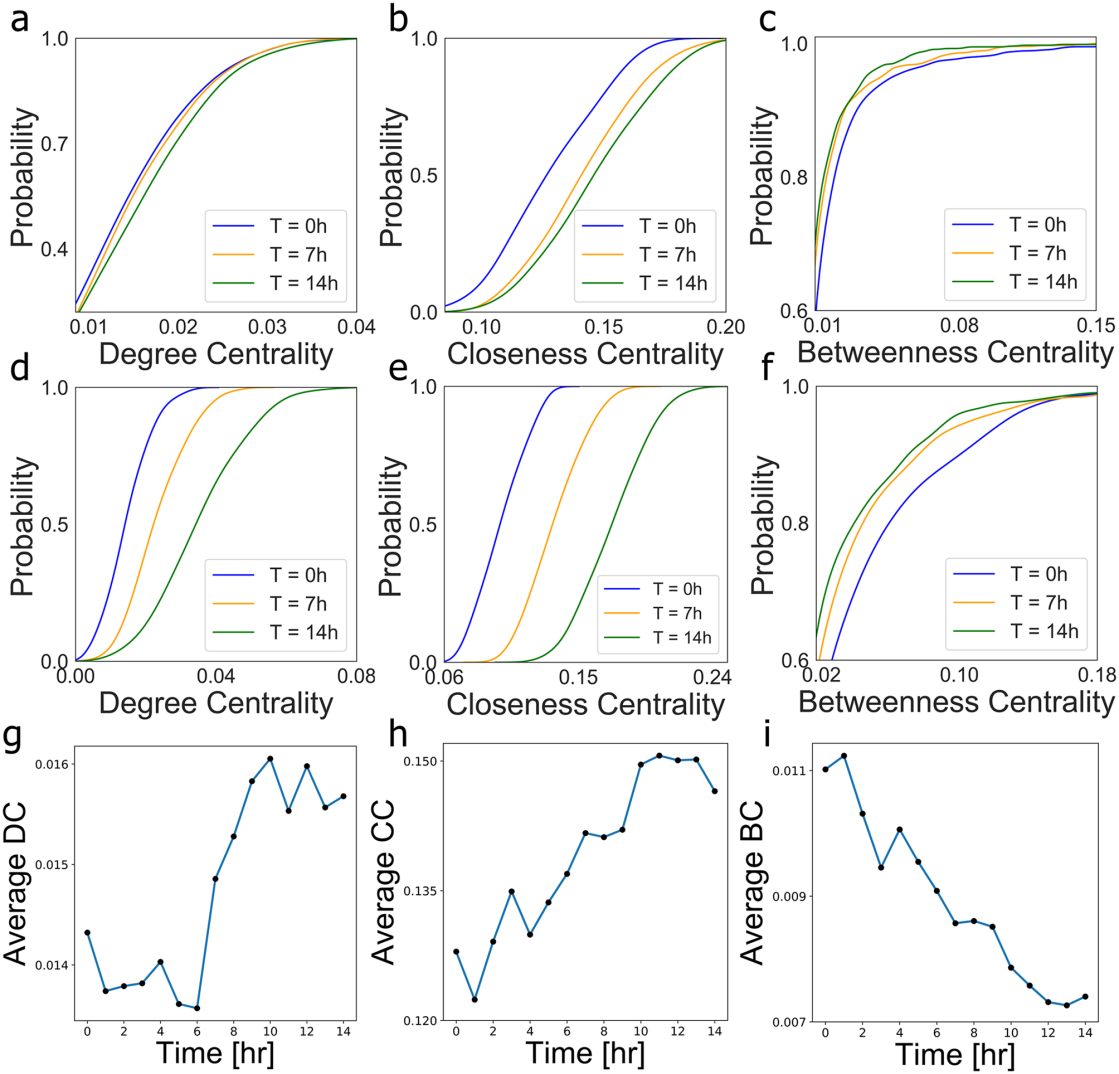
Investigate the changes of degree-, closeness-, and betweenness-centrality in consecutive neuronal culture networks and neuronal culture cluster networks. The CDF curves of the degree centrality (**a**), closeness centrality (**b**) and betweenness centrality (**c**) for neuronal culture networks for three times t = 0, 7, and 14 h. The CDF curves of the degree centrality (**d**), closeness centrality (**e**) and betweenness centrality (**f**) for neuronal culture cluster networks for three times t = 0, 7, and 14 h. The average degree centrality (**g**), average closeness centrality (**h**) and average betweenness centrality (**i**) for neuronal culture networks for 15 time points within the 14 h experiment.

**Figure 4:**
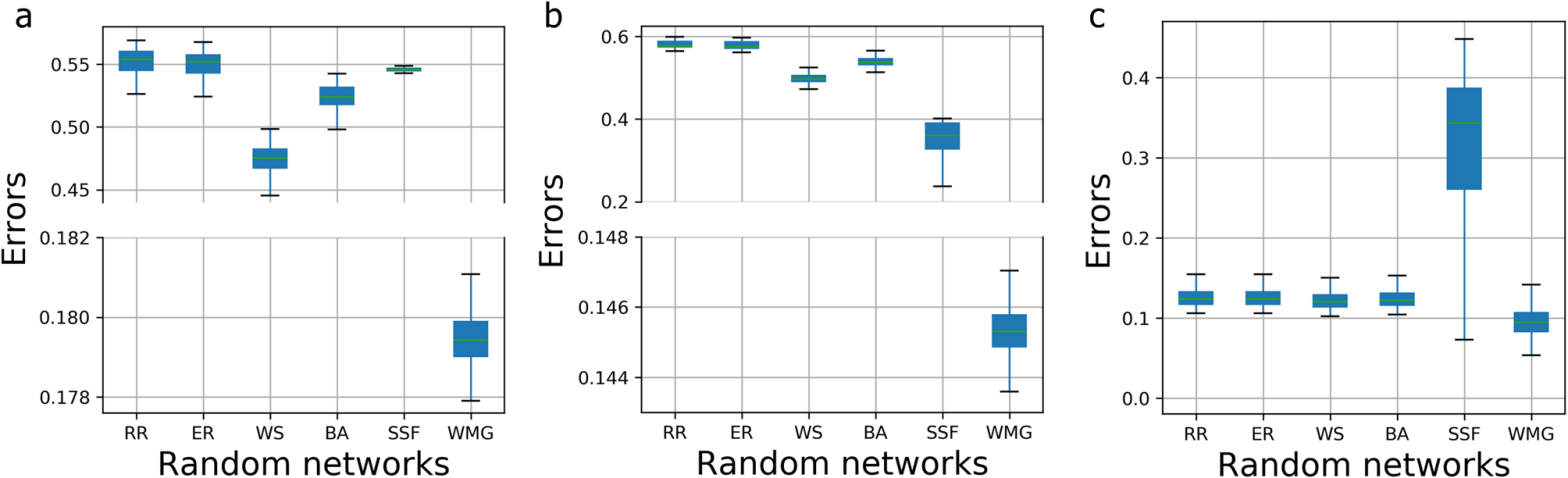
Comparison of clustering indices between the neuronal culture networks and model-based randomly constructed networks of the same size. (**a**) The comparison (errors) in terms of the transitivity between the neuronal culture networks and the RR, ER, WS, BA, SSF, and WMG based generated networks (for each model we generated 1000 network realizations) for the 14 h experiment. (**b**) The comparison (errors) in terms of average clustering coefficient between the neuronal culture networks and the RR, ER, WS, BA, SSF, and WMG based constructed networks (for each model we generated 1,000 networks) within 14 h. (**c**) The comparison (errors) in terms of average square clustering coefficient between the neuronal culture networks and the RR, ER, WS, BA, SSF, and WMG based networks during the 14 h experiment.

Figure 3 illustrates the cumulative distribution function (CDF) curves of the degree centrality, closeness centrality and betweenness centrality estimated for three consecutive snapshots of a single brain-derived neuronal culture network and a single brain-derived neuronal culture cluster network (Supplementary Figs. S2, S3 present the histograms and smoothed curves about the degree-, closeness, and betweenness centrality for neuronal culture network and neuronal culture cluster network). For instance, over the course of the experiment, the CDF curves of the degree centrality in Fig. 3a of the microscopic neuronal culture networks exhibit a shift towards higher degree centrality values. This is best reflected in Fig. 3g, where the average degree centrality shows an increasing trend. At a higher scale of a network of neuronal clusters and the same three time points, the CDF curves of degree centrality exhibit a more pronounced shift to higher values (see Fig. 3d). The higher degree centrality values are the higher the chance of nodes to receive the information passed over the network. These results demonstrate that the networks of neurons and neuronal clusters tend to optimize the degree centrality and support higher information transmission across the network over time. Neuronal culture networks achieve this increase in degree centrality by growing connections, while the network of neuronal clusters increases their degree centrality through the merging of clusters and connection inheritance.

Along the same lines, Fig. 3b for the network of neurons and Fig. 3e for the network of neuronal clusters show that the CDF curves of closeness centrality is shifting to the right (higher values). This trend can also be observed in Fig. 3h where the average closeness centrality has an upward tendency. The higher the closeness centrality of a node is the less time it takes for this node to reach all other nodes. Consequently, these results show that the network of neurons and the network of neuronal clusters tend to optimize the closeness centrality and minimize the information transmission latency. By comparing the dynamics of the network of neurons with that of the network of neuronal clusters, we observe a doubling effect for the magnitude of the location of the peak in the closeness centrality CDF curves.

The analysis of the betweenness centrality CDF curves and the average betweenness centrality shows a decreasing tendency for both the networks of neurons (Fig. 3c) and the network of neuronal clusters (Fig. 3f). A lower node betweenness centrality means that the node appears fewer times along the shortest path among all network nodes. Of note, during the course of the experiment, we observe that some neurons die and are deleted from the network. If a neuron with a high betweenness centrality is removed from the network, then the network has a higher chance of becoming disconnected. However, since the average betweenness centrality is decreasing over time, the dying neurons have a lower probability of causing network disconnection. Thus, we conclude that the networks of neurons and neuronal clusters tend to minimize the betweenness centrality, which can increase the robustness of the network against cascading failures. In summary, the analysis of degree centrality, closeness centrality and betweenness centrality shows that the networks of neurons and neuronal clusters tend to optimize the network information transfer.

**Figure 5:**
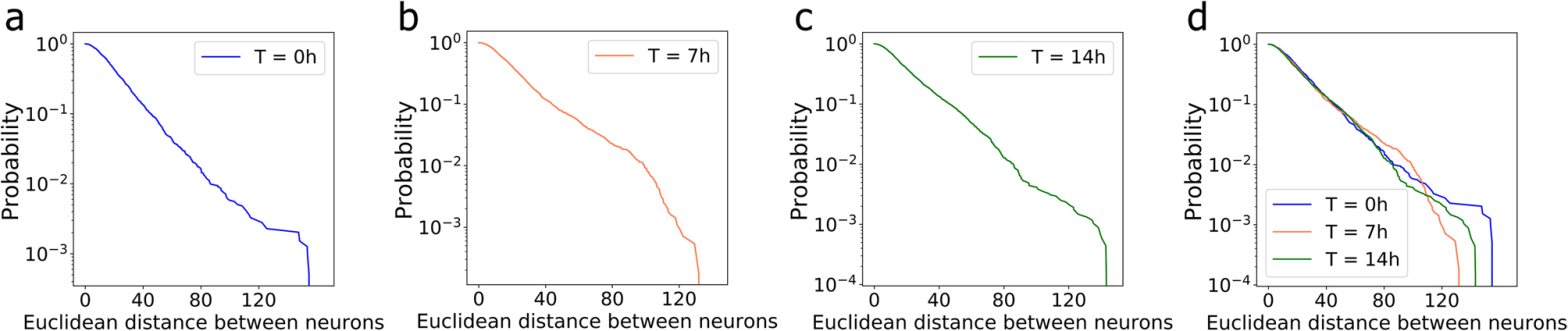
Variations of interconnections between two neighboring neurons. The exceedance probability for the length of connections between two neurons (i.e., the probability of observing the length of a connection between two neurons exceeding a certain threshold) at the start of the experiment t = 0 h (**a**), after 7 h (**b**), at the end of the experiments after 14 h (**c**), and the comparison of (**a**–**c**) is shown in (**d**).

**Figure 6:**
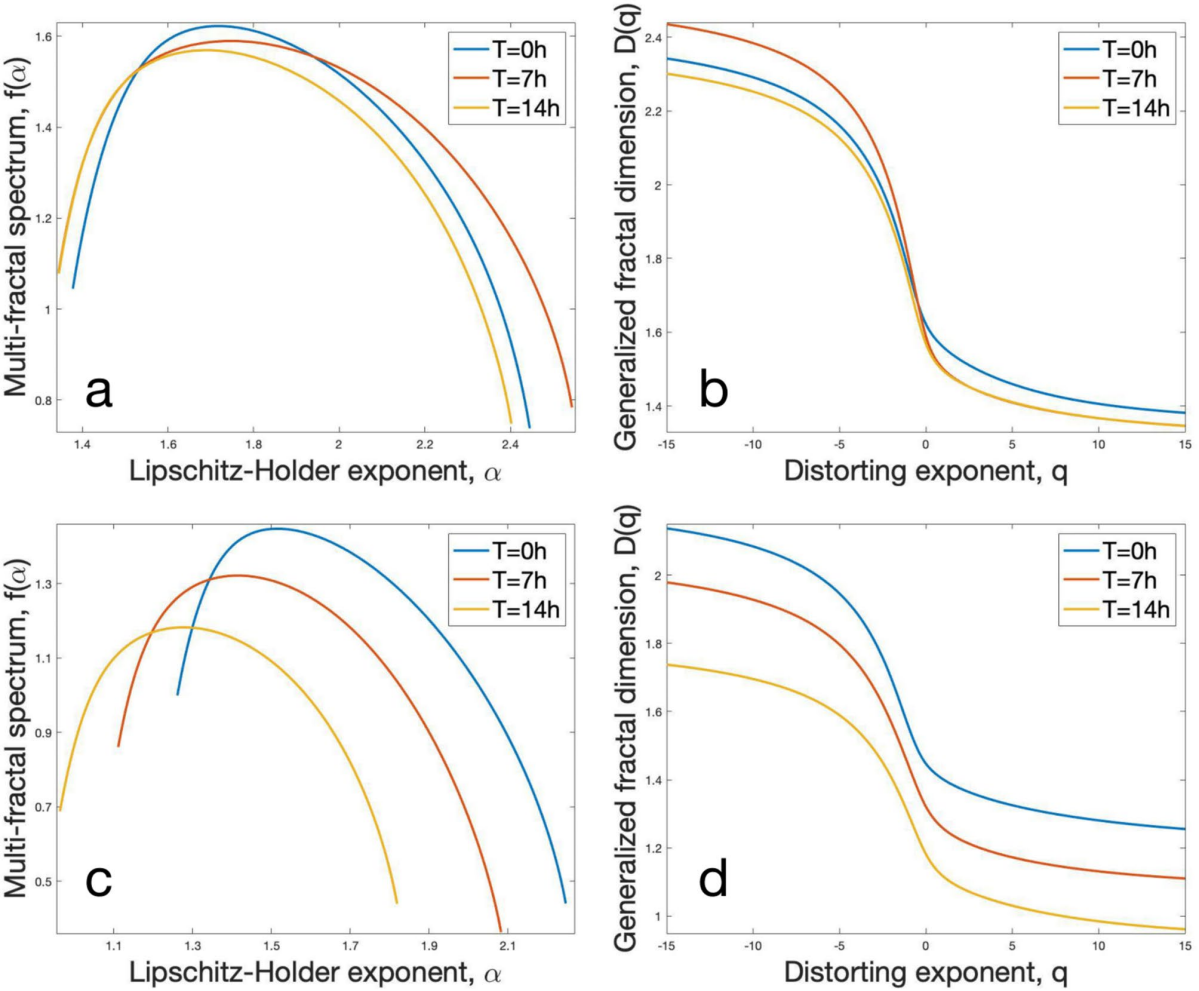
Multifractal analysis of neuronal culture networks and neuronal culture cluster networks. (**a**) Multifractal spectrum $f(\alpha )$ as a function of Lipschitz–Holder exponent $\alpha$ for neuronal culture networks in t = 0, 7, and 14 h. (**b**) Generalized fractal dimension *D*(*q*) as a function of q-th order moment for neuronal culture networks in t = 0, 7, and 14 h. (**c**) Multifractal spectrum $f(\alpha )$ as a function of Lipschitz–Holder exponent $\alpha$ for neuronal culture cluster networks in t = 0, 7, and 14 h. (**d**) Generalized fractal dimension D(q) as a function of q-th order moment for neuronal culture cluster networks in t = 0, 7, and 14 h.

### Networks of neurons and networks of neuronal clusters display a significant clustering phenomenon compared to state-of-the-art complex network models

In this section, we investigate whether the network of neurons can be well described by existing complex network models (i.e., Random Regular (RR)^13^, Erdos-Renyi (ER)^14^, Watts-Strogatz (WS)^15^, Barabasi-Albert (BA)^16^, Spatial Scale-Free model (SSF)^22,23^ and Weighted Multifractal Graph (WMG)^24^) by generating artificial networks of the same size (in terms of the number of nodes and edges) according to these models for all the considered time points (within 14 hours) and computing their transitivity, clustering coefficient, and the square of the clustering coefficient metrics (see Fig. 4). RR network is defined as a random d-degree network on **n** nodes; ER network is $G_{(n,p)}$ model, where **n** is the number of nodes, **p** is the linking probability; WS is a random network model which has small-world network properties; BA model generates scale-free networks characterized by a power law degree distribution; SSF model exhibits spatial scale-free networks where the probability of the incoming node *i* setting up a connection with an existing node *j* is $p_{i\rightarrow j} \propto k_{j}exp(-d_{ij}/r_{c})$ where $d_{ij}$ is the distance between *i* and *j*, $k_{j}$ is the degree of node *j* and $r_{c}$ is the control parameter. Besides these well-known complex network models, since the neuronal culture network and neuronal culture cluster network possess multifractal characteristics (for details on this conclusion, see “Results” section “Neuronal culture networks and neuronal culture cluster networks possess multifractal characteristics” and “Methods” section “Multifractal analysis”), we also investigated whether the WMG model can provide a better fit for the considered neuronal culture networks. The WMG model captures and generates weighted multifractal networks by mapping from recursively constructed measures of linking probability. The transitivity **T** of a graph is based on the relative number of triangles in the graph, compared to the total number of connected triples of nodes (also known as the global clustering coefficient). The clustering coefficient measures the degree to which the network nodes connect to each other. The square of the clustering coefficient quantifies the cliquishness in bipartite networks where triangles are absent (for details on the transitivity, clustering coefficient, and square of the clustering coefficient, see “Methods” section “Clustering coefficient”). We investigate the clustering coefficient because we observe that the neurons tend to organize into various communities over time (see Fig. 1j–l), in this way, it is likely that the neuronal culture networks have “small world”^15^ properties. As one can observe from Fig. 4, the values of the transitivity and clustering coefficient metrics for the network of neurons are significantly higher than those corresponding to the artificially generated networks corresponding to the first five above-mentioned models (To compare the biological networks against existing network models, for each time point, we compute the average and the 95% confidence intervals of the transitivity, clustering coefficient and averaged square clustering coefficient from 1,000 artificially generated network realizations). Alternatively stated, the neurons tend to form communities with very different topological structures than the well-known RR, ER, WS, BA, or the SSF models. In contrast, the fitting of the WMG model shows smaller errors in terms of the transitivity, clustering coefficient and averaged square clustering coefficient metrics.

After investigating the neuronal culture network structure characteristic, we analyze the spatial organization or metric correlations of neuronal culture networks based on the topology. We measure the functional relationship between the Euclidean distance of neighboring neurons and the number of interconnections among them. In Fig. 5, we calculate the probability of observing the length of a connection between two neurons exceeding a certain threshold (also called exceedance probability) for different timestamps (t = 0 h, t = 7 h and t = 14 h). We find that larger Euclidean distances (threshold) have lower probabilities. These results indicate that the physically closed neurons have more connections than the physically distant ones. Furthermore, we observe that the distance between arbitrary two neurons in the same community decreases, while the number of edges in each community increases. These observations also corroborate with the conclusions drawn from analyzing the degree centrality and closeness centrality. In summary, we conclude that the network of neurons (1) possesses a network generator that is different from the RR, ER, SW, BA, and SSF models, (2) has more interconnections between physically closer neurons, (3) has the tendency to self-optimize in order to enable and support higher, faster and more robust information transmission, and (4) exhibits multifractal topological characteristics.

**Table 2:** The parameters of multifractal spectrum.

Parameters	Neuronal culture networks			Neuronal culture cluster networks		
	Start	Middle	End	Start	Middle	End
$f(\alpha )_{max}$	1.6220	1.5892	1.5690	1.4472	1.3213	1.1821
$\alpha _{max} - \alpha _{min}$	1.0661	1.1974	1.0561	0.9849	0.9708	0.8548
$f(\alpha )_{max}-f(\alpha )_{min}$	0.8840	0.8057	0.8211	1.0071	0.9581	0.7426

### Neuronal culture networks and neuronal culture cluster networks possess multifractal characteristics

Previous works^25–27^ have argued that the brain intelligence is correlated with the regional gray matter, volume, tissue, and microstructure of white matter. Here, we adopt an alternative topological perspective to the correspondence between neuronal connectivity complexity and intelligence and analyze the multifractal characteristics of neuronal culture networks. To comprehensively observe the structural complexity and heterogeneity of neuronal culture networks and neuronal culture cluster networks, we use the finite box-covering algorithm^28^ and estimate their multifractal spectrum and generalized fractal dimension (for details on the multifractal analysis and box-covering algorithm, see “Method” section “Multifractal analysis”). Multifractal analysis (MFA) applies a distorting exponent *q* to the probability measure at different observation scales and can quantify the structural characteristics of networks by comparing how the network behaves at each distortion. MFA provides information about the heterogeneous self-similarity of our networks and can help us identify changes in their topological heterogeneity over time. By observing the multifractal spectrum $f(\alpha )$ under different Lipschitz–Holder exponent $\alpha$, we can capture the variation in scaling behaviors of different subcomponents of the network. Equivalently, this variation could be observed by learning the generalized fractal dimension *D*(*q*) under the order *q*. In multifractal spectrum, the larger the $\alpha$, the higher density of the self-similar structure in the network; the larger the $f(\alpha )$, the larger the amount of the self-similar structures in the network; the larger the width ($\alpha _{max} - \alpha _{min}$), the more diverse the fractal structure in the network.

Applying the MFA for the neuronal culture network (i.e., Fig. 6a,b, Table 2) and the neuronal culture cluster network (i.e., Fig. 6c,d, Table 2) across time, we observe: (i) Fig. 6 shows that the neuronal culture networks and the neuronal culture cluster networks possess multifractal properties. By comparing their multifractal spectrum parameters summarized in Table 2, we conclude that the $f(\alpha )_{max}$ and the width of the spectrum of the neuronal culture network are larger than those of the neuronal culture cluster network. Consequently, the neuronal culture networks have stronger multifractality, which means stronger heterogeneity and higher complexity. (ii) From Fig. 6a,b, we can see that although the number of edges of the neuronal culture network increase across time, its multifractal spectrum and the generalized fractal dimension have only small changes without monotonic variation with time. The spectrum has no tendency to move over time, which shows the common self-similar structures of the neuronal culture network do not change. Therefore we can conclude that our neuronal culture network has a relatively stable multifractal structure, which means even if neurons generate new connections over time, the self-similar structures of the neuronal culture network do not change much. (iii) From Fig. 6c,d, we can see different trends from the neuronal culture network. The multifractal spectrum and the generalized fractal dimension of the neuronal culture cluster network exhibit a monotonic pattern over time. The result shows that the multifractal spectrum moves down to the left, which means the common self-similar structures of the neuronal culture cluster network become less dense. The width of the spectrum and the generalized fractal dimension decrease across time, which means the self-similar structures become more concentrated so the diversity of the network structure decreases with time. This is because in the neuronal culture cluster network, the clusters move and sometimes join to form a larger cluster. The continuous merger behavior will bring structural changes that reduce the heterogeneity of the neuronal culture cluster network as our results show.

## Discussion

By adopting a complex networks characterization, we find that brain-derived neuronal culture networks and neuronal culture cluster networks of rats and mice exhibit a network flow self-optimization phenomenon (i.e., higher information transmission, latency reduction, and robustness maximization over time) either by growing connections or via the merging of neuronal clusters. This analysis complements and contributes to earlier studies that showed the existence of self-organized criticality, of a small-world state and that higher clustering leads to spontaneous bursting in parts of the neuronal culture networks^10,11^. Future work should investigate whether the self-organized criticality is goal-driven and contributes to the observed self-optimization phenomenon. Furthermore, we concluded that neuronal interconnection architecture displays assortative behavior. To elucidate the mechanisms by which neurons create or suppress connections to enable communication in brain networks and understand their role in learning, cognition, and creative behavior, future studies should combine the complex sensing approach^12^ of probing the neuron and glial cell activity coupling with network science concepts and tools presented in this study. In addition, our clustering analysis demonstrates that the network model characterizing the brain-derived neuronal culture networks does not fit the Random Regular (RR)^13^, Erdos-Renyi (ER)^14^, Watts-Strogatz (WS)^15^, Barabasi-Albert (BA)^16^, and Spatial Scale-free (SSF) network models^22,23^. In contrast, the weighted multifractal graph model^24^ provides the best fit (smallest error) in terms of matching the clustering, transitivity, and square clustering coefficients. Finally, by analyzing the spatial properties associated with the topology of the monitored neuronal culture networks we observe that closer neurons have more interconnections among them than the distant ones.

Current neuroscience studies^29,30^ discuss the importance of investigating the in vitro neuronal cultures as an efficient system to model the neural activity, as well as, the role of understanding the spatial embedding and metric correlations on connectivity and activity in neuronal culture networks^31^. Along these lines, our proposed combined network science framework and image processing tool can be further employed for analyzing the interactions and metabolic coupling between neurons and glial cells (e.g., astrocytes) either via fMRI sensing^12^ or an enhanced quantitative phase imaging approach used in this work for live monitoring of neurons and glia. With the goal of investigating the pulsation in vitro neuronal cultures, Orlandi et al.^29^ showed that neuronal spiking behavior can originate from a random set of spatial locations specific to each culture and is modulated by a nontrivial interdependence between topology and neural dynamics. To study the spatial arrangement of neurons in neuronal cultures, a random field Ising inspired model^32^ showed that metric correlations dominate the neuronal topological properties. Tibau et al.^33^ extracted the effective connectivity of neuronal cultures from the spontaneous activity of calcium fluorescence imaging recordings and observed an increase in average connectivity over time and various degrees of assortativity. This body of work suggests that the spontaneous activity in the mammalian brain plays a fundamental role in brain development, information transmission, and communication of different brain regions and provides a new research direction to investigate the functional relationship between the evolution of the neuronal culture networks (with multifractal characteristics) and neuronal spiking activities.

In this work, we investigated the mathematical properties of brain-derived neuronal culture networks and brain-derived neuronal culture cluster networks (by precisely locating and detecting each axon and dendrite within 0.03nm optical path-length accuracy^34^) which provides a way to analyze the spontaneous evolution of the neuronal cultures in the early stages (i.e., 14 h). Furthermore, future studies should characterize and distinguish between healthy and unhealthy behavior (e.g., glioblastoma/brain tumor) of neurons as well as identifying the degree of toxicity of cultures. Moreover, future mathematical analysis of neuronal culture networks can also help us understand how neurons connect to guide the information flow as we recall the past, envision the future, or make social inferences, model the perception, inference, generalization and decision making. Lastly, by explaining the mechanisms of cognitive control emerging from multiscale neuronal culture networks, we can identify new biological inspired strategies for designing deep learning architectures.

## Methods

### Sample preparation

Neural clusters were prepared from mouse neurons and neural networks were prepared from rat neurons. Neurons harvested from B6/J mice were thawed and plated on poly-d-lysine-coated glass-bottom petri dishes. Low-density cultures (65 cells per mm^2^) were grown at 37 °C, in the presence of 5% CO2, in Neurobasal growth medium supplemented with B-27, 1% 200 mM glutamine and 1% penicillin/streptomycin. All reagents were sourced from Invitrogen (Thermo Fisher Scientific). Half the media was aspirated twice a week and replaced with fresh maintenance media warmed to 37 °C. Live-cell imaging took place three days in vitro^35^.

Dissected cortical tissue from AGE rats was dissociated in 3 mg/mL protease 23 (Sigma P4032) in 1 × slice dissection solution (pH 7.4). Primary neurons were grown on Poly-d-Lysine (Advanced BioMatrix 5049-50) treated glass-bottom dishes (Cellvis, P06-14-0-N). Cells were grown in maintenance media for 10 days before time-lapse microscopy. We observe a density of approximately 800 cells per mm^2^.

### Microscopy

Spatial light interference microscopy (SLIM) is an optical microscopy technique that can capture the evolution of living neurons^34^. Neurons are particularly challenging to image, as complex phenotypes such as arborization are adversely modulated by phototoxicity. A higher resolution SLIM imaging method could decouples amplitude artifacts from high detailed cellular information. When imaging neural networks, we attempt to ameliorate phototoxicity concerns by reducing the illumination intensity (Thorlabs MCWHL5, 30 milliamps, 3% of total power) and average over several images following the hybrid denoising scheme in^36^. To boost the sensitivity of our measurements, we choose to use Spatial Light Interference Microscopy (SLIM Pro, Phi Optics) which is particularly well suited to imaging the fine details found in neuronal arbors^35^.

### Cell segmentation and neural tracing

The difference between neuronal culture networks and neuronal culture cluster networks is the definition of the node. In the neuronal culture network, the node is defined as a single neuron and in the neuronal culture cluster network, the node is defined as a cluster of neurons. To extract neuron connection information and set up neuronal and neuronal culture cluster networks from our brain neurons dataset, our process has the following steps: (1) *Extracting valid data* We pick out the clear parts from all our neuron pictures via a machine learning approach (i.e., LeNet model). We randomly pick $20\%$ figures from our dataset, check the resolution of each figures, and label these figures with 0s (representing dim) and 1s (representing clear) to generate our training set. We construct a convolutional neural network (CNN) model called LeNet^37^ and train it with the training set. Then we test on the remaining $80\%$ figures. Finally, based on the testing result from our LeNet model, we select the parts with relatively high resolution in all time points for image processing. (2) *Differentiating the debris and neurons* Since neurons (or neuron clusters) in the picture can be detected from their bright round shape, we set the minimum size of neurons as follows. For the rat brain image dataset and the mouse brain image dataset, the minimum size of neurons and neuron clusters are set as 600 pixels and 800 pixels. If the size of a bright round shape is less than the threshold, it is defined as debris. (3) *Filling gaps between neurites* There are many gaps existing between neurites caused by insufficient sharpness of the original neuron pictures or by synaptic cleft, which would disconnect the neurites or connectivities between neurons. Therefore, we propose a terminal detection algorithm (via detecting the number and location of pixels) and mark all the terminals from our database. If a neuron of neurite lays around a marked terminal, we will fill in this gap and consider this neurite connects with the marked terminal. (4) *Tracing the neurites* We develop a tracing algorithm (using a steerable filter) to trace the neurites. Considering one neuron as the source node and track its neurites pixels by pixels. If a neurite connects with another neuron, these two neurons are recorded as connected. By repeating the tracing algorithm in relation to all neurons, we can achieve neuronal and neuronal culture cluster networks.

### Networks centrality

In this paper, network centralities are analyzed by the NetworkX package in Python. The networks centrality measures the importance of a node across a heterogeneous complex network. We briefly introduced the degree-, closeness-, and betweenness- centrality as follows:

Degree centrality^38^ of node *v* is defined as:

1 $$\begin{aligned} Degree(v)=deg(v) \end{aligned}$$

where *deg*(*v*) is the number of links upon node *v*. The node-to-node degree distribution means the quotient between degree distribution of *u* and *v* (assume a edge *e*(*u*, *v*), where *u* is the source node and *v* is the destination node).

Closeness centrality^39^ of a node quantifies the average length of the shortest path between the target node and all other nodes in the graph and encodes information about the information transmission latency across a specific network topology. It can be defined as:

2 $$\begin{aligned} Closeness(v)=\frac{1}{\sum _{u}d(u,v)} \end{aligned}$$

where *d*(*u*, *v*) is the distance between node *u* and node *v*.

Betweenness centrality^40^ is generally used to measure the number of time a node appears along the shortest paths between two other randomly picked nodes. It can be defined as:

3 $$\begin{aligned} Betweenness(v)=\sum _{s\ne v\ne t\in V}\frac{\sigma _{st}{(v)}}{\sigma_{st}} \end{aligned}$$

where $\sigma_{st}$ is the number of shortest paths between node *s* and *t* and $\sigma_{st}(v)$ is the number of these shortest paths which pass through the node *v*.

### Clustering coefficient

In this paper, these network clustering coefficients are analyzed by the NetworkX package in Python. The clustering coefficient measures the degree of which degree in a complex network tend to cluster together.

Transitivity also called global clustering coefficient is based on triplets of nodes. Triplet means a group of three nodes which are connected by two or three edges. It can be defined as:

4 $$\begin{aligned} C=\frac{3\times number\ of\ triangles}{number\ of\ all\ triplets} \end{aligned}$$

The clustering coefficient (or local clustering coefficient) measures how close /connected a node is to its neighbors and forming a clique (i.e., a complete graph). The clustering coefficient is defined as follows:

5 $$\begin{aligned} C(G)=\frac{1}{|V'|}\sum _{v\in V'}{c(v)} \end{aligned}$$

where $V'$ is the set of nodes (*v*) whose degree is larger or equal to 2. $c(v)=\delta (v)/\tau (v)$, where $\delta (v)$ is the number of triangles of node *v* and $\tau (v)$ is the number of all triplets of node *v*.

The squared clustering coefficient quantifies the cliquishness in bipartite networks (e.g. social network) where triangles are absent (the standard clustering coefficient is always zero). Similar with the triangles, the squares clustering coefficient is the rate between the number of squares and the total number of possible squares^41^. It can be defined as:

6 $$\begin{aligned} C_{4, mn}(i)=\frac{q_{imn}}{(k_{m}-\alpha _{imn})(k_{n}-\alpha _{imn})+q_{imn}} \end{aligned}$$

where $q_{imn}$ represents the number of neighbors of *m* and *n* (not considering node *i*); $\alpha _{imn}$ is represented as $\alpha _{imn}=1+q_{imn}+\theta _{mn}$; $\theta _{mn}$ is 1 if *m* and *n* are connected and 0 otherwise; $k_{i}$ is the number of neighbors for node *i*.

### Multifractal analysis^42^

Using the box-covering algorithm in a mono-fractal network, we can capture the relationship of *r* (e.g., the size of the box) and *M*(*r*) (the number of nodes in the box) as a power law of the form $M(r)\sim r^D$, where *D* is the fractal dimension (a real-valued number representing the mono-fractal feature of the network). Multifractals could be considered as the superposition of multiple mono-fractals, and we use the finite box-covering algorithm^28^ to study the localized and heterogeneous self-similarity of networks. To capture the multifractal features in networks, the distortion factor *q* was introduced to distinguish the details of different fractal structures. Then we can capture the multifractality of the network by learning a generalized fractal dimension *D*(*q*) under different distortion factors *q*. In this way, the number of nodes in the *i*th box scales as $M_i(r)\sim r^{\alpha _i}$ and the number of boxes with the same $\alpha$ scales as $N(\alpha )\sim r^{-f(\alpha )}$, where $\alpha$ is the Holder exponent. The relationship between the pair of (*D*(*q*), *q*) and $(f(\alpha ),\alpha )$is decided by the Legendre transformation as:

7 $$\begin{aligned} \alpha= & {} \frac{d}{dq}[(q-1)D(q)]\ \end{aligned}$$

8 $$\begin{aligned} f(\alpha )= & {} q\alpha -(q-1)D(q)\ \end{aligned}$$

Thus, we can use Eqs. (7) and (8) to calculate the multifractal spectrum $f(\alpha )$, therefore, we can analysis the multifractality of the network by observing the spectrum.

### Ethical approval

All animal procedures were carried out per approved protocols from the Institutional Animal Care and Use Committees (IACUC) at University of Nebraska Medical Center and University of Illinois Urbana Champaign, and in accordance with the recommendations in the Guide for the Care and Use of Laboratory Animals of the National Institutes of Health. (Animal Assurance PHS: #A3294-01, Protocol Number: 10-033-08-EP).

## Supplementary information

Supplementary Information

Supplementary Legends

Supplementary Video 1.

Supplementary Video 2.

## Data Availability

The datasets analysed during the current study are available from the corresponding author on reasonable request.
