# Supplementary material for: Network science characteristics of brain-derived neuronal cultures deciphered from quantitative phase imaging data
Source: Sci Rep. 2020 Sep 15;10:15078. doi: 10.1038/s41598-020-72013-7 (PMC7492189; doi:10.1038/s41598-020-72013-7)
Supplement: Supplementary file 1 — Supplementary Information [file 41598_2020_72013_MOESM1_ESM.pdf]

Supplementary Material for Network Science Characteristics of Brain-derived Neuronal Cultures Deciphered from Quantitative Phase Imaging Data

Chenzhong Yin[1,+], Xiongye Xiao[1,+], Valeriu Balaban[1,+], Mikhail E Kandel[2,+], Young Jae Lee[2,3], Gabriel Popescu[2], and Paul Bogdan[1*]

[1]Ming Hsieh Department of Electrical and Computer Engineering, University of Southern California, Los Angeles, CA 90007, USA.
[2]Beckman Institute for Advanced Science and Technology, Department of Electrical and Computer Engineering, University of Illinois at Urbana-Champaign, Champaign, IL 61801, USA.
[3]Neuroscience Program, University of Illinois at Urbana Champaign, 208 N Wright St., Urbana, IL 61801, USA
[*]Correspondence and requests for materials should be addressed to P.B. (email: pbogdan@usc.edu)
[+]These authors contributed equally to this work

ABSTRACT

Understanding the mechanisms by which neurons create or suppress connections to enable communication in brain-derived neuronal cultures can inform how learning, cognition and creative behavior emerge. While prior studies have shown that neuronal cultures possess self-organizing criticality properties, we further demonstrate that in vitro brain-derived neuronal cultures exhibit a self-optimization phenomenon. More precisely, we analyze the multiscale neural growth data obtained from label-free quantitative microscopic imaging experiments and reconstruct the in vitro neuronal (microscale) networks and neuronal cluster (mesoscale) networks. We investigate the structure and evolution of neuronal and neuron cluster networks by estimating the importance of each network node and their information flow. By analyzing the degree-, closeness-, and betweenness-centrality, the node-to-node degree distribution (informing on neuronal interconnection phenomena), the clustering coefficient/transitivity (assessing the "small-world" properties), and the multifractal spectrum, we demonstrate that murine neurons exhibit self-optimizing behavior over time with topological characteristics distinct from existing complex network models. The time-evolving interconnection among murine neurons optimizes the network information flow, network robustness and self-organization degree. These findings have complex implications for modeling neuronal cultures and potentially on how to design biological inspired artificial intelligence.

Supplementary Figures

Our supplementary file displays our experimental figures which provides more detailed information about our figures in the manuscript. Fig. S1 expresses an example of how we construct the neuronal networks and neuronal cluster networks. We also exhibit two example videos to present how our algorithm works (Video S1 is for neuronal culture network and Video S2 is for neuronal culture cluster network). Tables S1 and S2 illustrate the degree-, closeness-, betweenness centrality for the artificial network (Fig. 2(a) in the manuscript) without and with additional connections in order to mimic the fluctuation of network centralities in neuronal networks and neuronal cluster networks. Fig. S2 illustrates the histograms of the degree centrality, closeness centrality and betweenness centrality estimated for three consecutive snapshots of the neuronal network and neuronal cluster network. Figs. S3 shows the smoothed curves of Fig. S2. By analyzing Figs. S2 and S3, we can easily find that the degree centrality and closeness centrality for neuronal networks and neuronal cluster networks are increasing over time and the betweenness centrality for the neuronal networks and neuronal cluster networks is decreasing over time. These phenomenon can support our conclusion that the networks of neurons and neuronal clusters tend to maximize their degree- and closeness centrality and minimize their betweenness, which illustrates that neuronal and neuronal cluster networks tend to optimize the network information transfer. Fig. S4 demonstrates a comparison of clustering indices (transitivity, clustering coefficient, and square clustering coefficient) between the neuronal networks and randomly constructed networks (i.e., random regular (RR), Erdos-Renyi (ER), Watts-Strogatz (WS), and Barabasi-Albert (BA)) of the same size. As we can observe from Fig. S4, the values of transitivity, clustering coefficient, and square clustering coefficient for the neuronal networks are higher than the four random network models (RR, ER, WS, BA). This observation illustrates that networks of neurons possess a network generator that is different from the RR, ER, SW, and BA models.

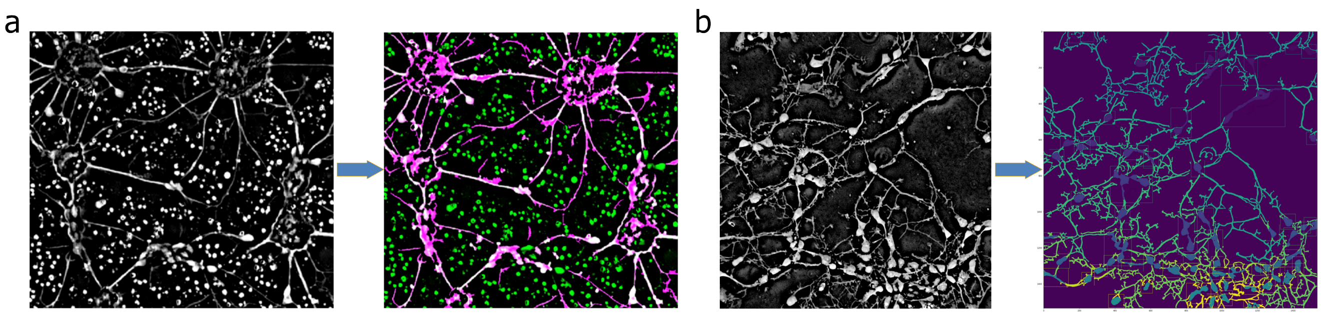

Figure S1. Layouts for sample neuronal network and neuronal cluster network. (a) Layout for sample neuronal cluster network after executing our segmentation algorithm, where the purple parts represent the neuron clusters and neurites and green nodes represent debris. (b) Layout for sample neuron network after executing our segmentation algorithm, where different color represents the identification of each neuron and neurite.

Table S1. Network centralities for artificial network (without additional connections)

Nodes	1	2	3	4	5	6	7	8	9	10	11	12	13	14
Degree Centrality	0.2308	0.2308	0.2308	0.3846	0.2308	0.3077	0.3077	0.1538	0.1538	0.2308	0.2308	0.2308	0.2308	0.2308
Closeness Centrality	0.3170	0.2766	0.3714	0.3421	0.4333	0.3333	0.3939	0.3023	0.4063	0.2955	0.2453	0.3611	0.2453	0.2955
Betweenness Centrality	0.0288	0.0107	0.1795	0.1132	0.5491	0.0609	0.2885	0.0000	0.5128	0.1282	0.0042	0.4658	0.0043	0.1282

Table S2. Network centralities for artificial network (without additional connections)

Nodes	1	2	3	4	5	6	7	8	9	10	11	12	13	14
Degree Centrality	0.3846	0.3846	0.3846	0.4615	0.4615	0.4615	0.4615	0.2308	0.2308	0.3077	0.3077	0.3846	0.3077	0.3077
Closeness Centrality	0.3611	0.4333	0.4333	0.4483	0.5417	0.4483	0.4483	0.4815	0.5417	0.3514	0.3514	0.4643	0.3514	0.3514
Betweenness Centrality	0.0051	0.0161	0.0161	0.0187	0.3678	0.0570	0.0570	0.0989	0.5171	0.0000	0.0000	0.4615	0.0000	0.0000

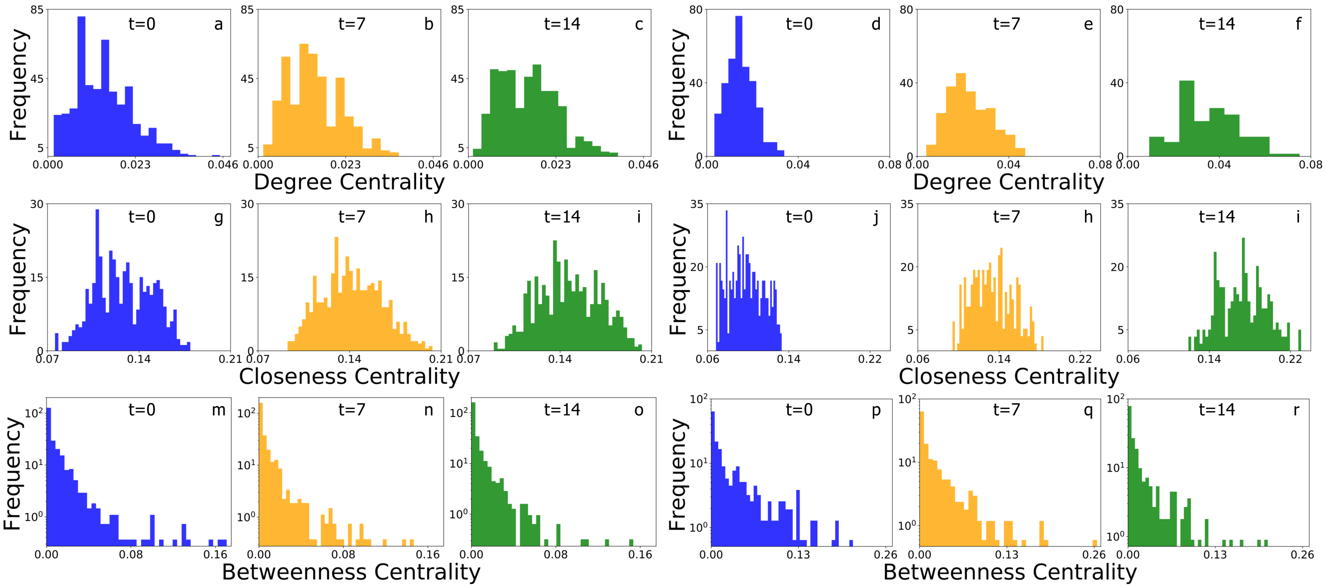

Figure S2. Investigate the changes of degree-, closeness-, and betweenness-centrality in consecutive neuronal networks and neuronal cluster networks. The histograms of the degree centrality (a), closeness centrality (b) and betweenness centrality (c) for neuronal networks for three times t = 0, 7, and 14 hours. The histograms of the degree centrality (d), closeness centrality (e) and betweenness centrality (f) for neuronal cluster networks for three times t = 0, 7, and 14 hours.

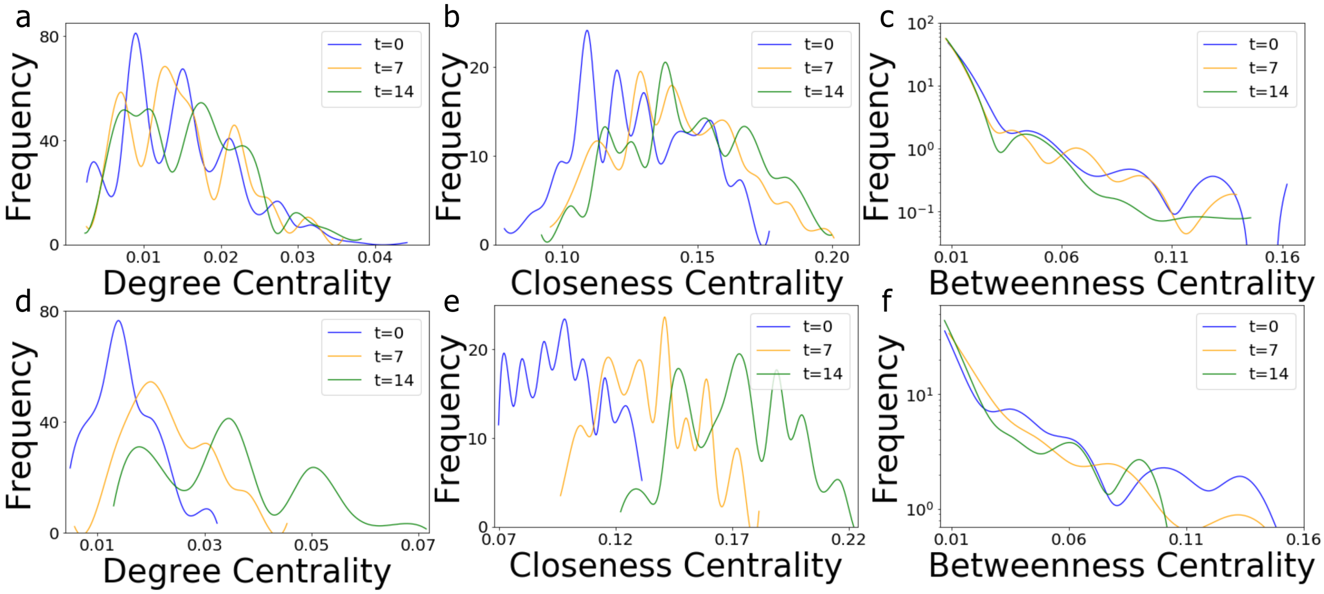

Figure S3. Investigate the shift of smooth curves for centrality metrics in neuronal networks and neuronal cluster networks. The smoothed curves of the degree centrality (a), closeness centrality (b) and betweenness centrality (c) for neuronal networks for three times t = 0, 7, and 14 hours. The smoothed curves of the degree centrality (d), closeness centrality (e) and betweenness centrality (f) for neuronal cluster networks for three times t = 0, 7, and 14 hours.

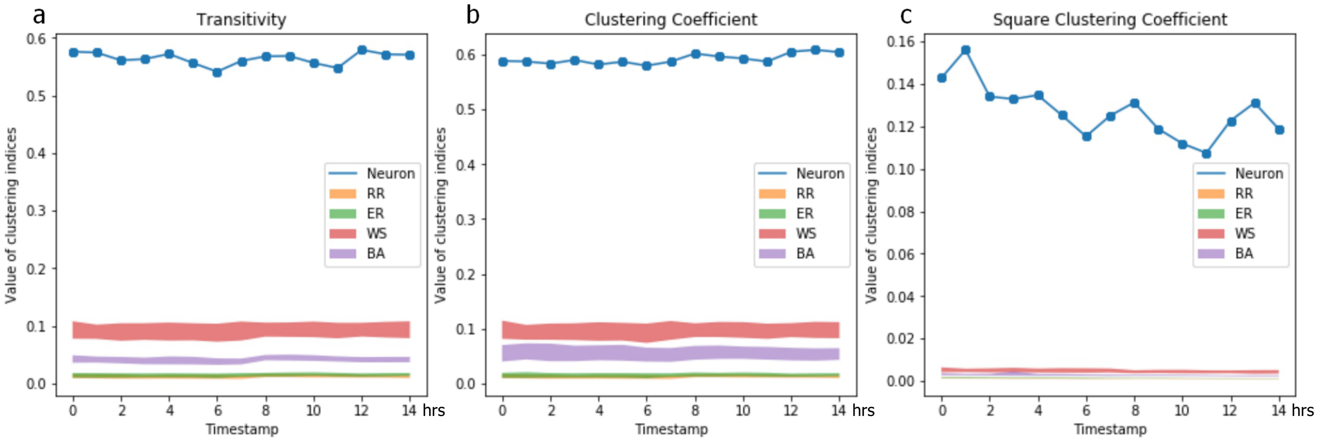

Figure S4. Comparison of clustering indices between the neuronal networks and model-based randomly constructed networks of the same size. (a) The comparison of transitivity between the neuronal network and random networks (each one has 1000 samples) within 14 hours. (b) The comparison of average clustering coefficient between the neuronal network and random networks (each one has 1000 samples) within 14 hours. (c) The comparison of average squares clustering coefficient between the neuronal network and random networks (each one has 1000 samples) within 14 hours.
