# Supplementary material for: Network science characteristics of brain-derived neuronal cultures deciphered from quantitative phase imaging data
Source: Sci Rep. 2020 Sep 15;10:15078. doi: 10.1038/s41598-020-72013-7 (PMC7492189; doi:10.1038/s41598-020-72013-7)
Supplement: Supplementary file 2 — Supplementary Legends [file 41598_2020_72013_MOESM2_ESM.docx]

**Video_S1.mp4**

This video depicts how our algorithm constructs an example neuronal culture network.

**Video_S2.mp4**

This video depicts how our algorithm constructs an example neuronal culture cluster network.
